# When dying does not go well: a qualitative study

**DOI:** 10.1186/s12904-024-01379-6

**Published:** 2024-03-09

**Authors:** Christof Breitsameter

**Affiliations:** Lehrstuhl für Moraltheologie, Katholisch-Theologische Fakultät, Ludwig-Maximilians-Universität München, Geschwister-Scholl-Platz 1, 80539 Munich, Germany

**Keywords:** Bad death, Idealization, Autonomy, Conflicts, Hospice, Palliative care unit

## Abstract

**Background:**

Several studies deal with the question of what constitutes a "satisfactory death". A smaller number of studies deal with unsatisfactory dying processes. And only a few shed light on unsatisfactory deaths that take place in hospices and palliative care units, which see themselves as places conducive to a "good" death. What also remains largely undiscussed are the ethical aspects that accompany the observation of an unsatisfactory course of death.

**Method:**

The research was carried out as an exploratory and qualitative study. The data collection and analysis were based on the methods of the "grounded theory".

**Results:**

Notions of a bad death are articulated here, though hardly by the affected persons and their relatives themselves, but rather by the professionals. Principally, descriptions of unsatisfactory dying processes refer to deficient success in symptom control, whereby the principle of autonomy is of particular importance. The focus here is not only on the needs of patients, but also on the needs of staff. The manifestation of such notions is related to the requirements arising from a practice that apparently evokes a need for accountability in the form of communicative reassurance.

**Conclusion:**

An idealised definition of "dying well" is in danger of losing sight of the contextual specifics of the practice involved, which can lead to ethically problematic situations.

**Supplementary Information:**

The online version contains supplementary material available at 10.1186/s12904-024-01379-6.

## Background

In recent decades, research on the concept of a good death (a better term would be "dying well") has been conducted repeatedly, often supported by qualitative and quantitative methods [1–3]. Such studies attempt to establish a plausible consensus in defining dying well by surveying indispensable features in end-of-life care [4–6]. Far less frequent are studies on an unsatisfactory dying process [7–12], especially concerning the course of death in hospices and palliative care units [13, 14]. It would be more important—especially in institutions where the idea or "ideology of a good death" [12] is particularly emphasized—to obtain information about when a death is considered unsatisfactory and to assess whether this judgment is justified. In addition, studies that focus on unsatisfactory deaths make little reference to the ethical aspects of this perception. We therefore addressed the question: How are dying processes that are not perceived as being satisfactory? What facets of an unsatisfactory death can be distinguished? And what can we learn from these descriptions regarding ethics?

## Methods

### Design

The research was carried out as an exploratory and qualitative study. The data collection and analysis were essentially based on the methods of the "grounded theory" form created by Barney Glaser and Anselm Strauss [15] and modified by Juliet Corbin and Anselm Strauss, [16] because it abstains from presupposed theoretical concepts, and therefore does not merely verify hypotheses, but generates them. The categories obtained in the research process were elaborated in terms of their theoretical properties and again tested in the field. The process of coding was carried out independently by the participating researchers, who have different professional backgrounds. The results were compared and discussed in regular meetings. The process of data collection was terminated after no additional categories could be extracted from the interviews, so we assume theoretical saturation with respect to the underlying research questions.

### Setting

Twenty five interviews were conducted at five hospices, 17 interviews in two palliative care units. 12 physicians, 27 nurses and 3 volunteers were interviewed.

### Participants

The selection process of study centers was focused on nationwide coverage (North, South, East, West). For the purpose of this article, however, we only evaluated the statements of the physicians, the nurses and the volunteers who work in hospices and palliative care units (see Table 1).

**Table 1:** Demographic details of participants

Age	19–62 years old (mean: 43 years old)
Gender	F = 27, M = 15
Institution	hospices = 25, palliative care units = 17
Participants from each institution	physicians = 12, nursing staff = 27, volunteers = 3

### Sampling

The study design is aiming at the observation of typical patterns in the data material. To achieve this, the sample must remain homogenous.

### Recruitment

For the selection of participants, the project was presented during the multi-professional staff meetings. Relevant staff members were asked to participate. There were no interactions with the participants prior to the study.

### Data collection

The data collection method was concentrated on problem-centered interviews. They interviews were based on thematic guidelines and were conducted in a semi-standardized form. The guidelines were designed for the different groups of people: There was one guide each for staff, and one for physicians. We asked the staff about the organization of everyday life, about the possibilities of contact with the patients/residents and relatives, about the needs and requirements of the patients/residents as well as about successful and problematic (end of life) care. We asked the physicians about the scope for shaping everyday practice, relationships with patients/residents, their wishes and fears, about their relationships with caregivers and relatives, as well as about successful and problematic (end of life) care. Most of the interviews were conducted one-on-one at the workplaces of the professionals between 2018 and 2019 by four different interviewers. They were digitally recorded and took 15 to 60 min (average length: 27,25 min).

### Analysis

**Fig. 1:**
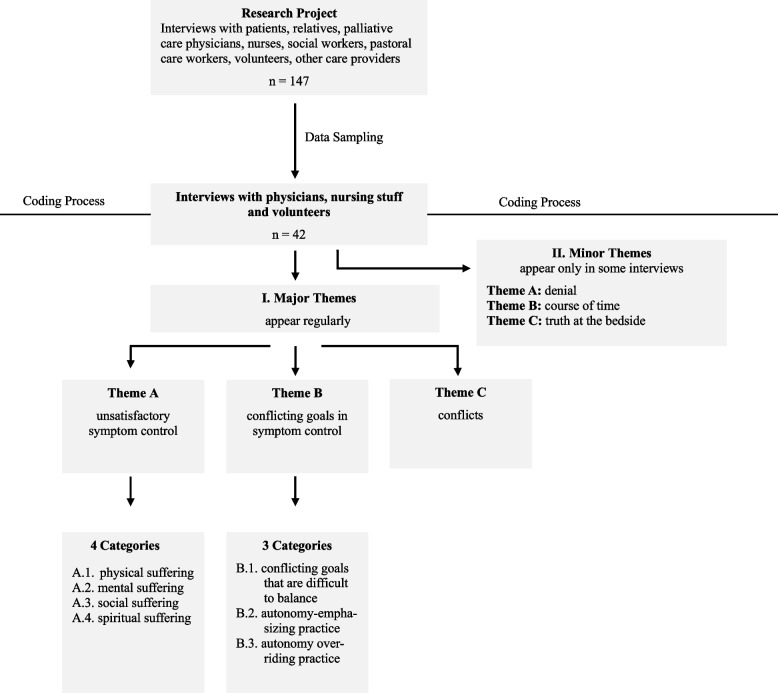
Flowchart of the research process

Recurring patterns and themes indicate an issue being addressed by the participants, that is a constitutive part of what we consider to be a perspective. We are interested in how different perspectives emerge and stabilize themselves through describing and addressing different problems. To achieve this, the transcripts of the interviews were analyzed with MAXQDA 2022. They were coded in a two-step process: The first step was an exploratory reading of the interviews, freely coding the material (open coding). After that, the different codes were reduced to major themes with corresponding categories and subcategories for each participant group (axial coding). Here, the definition of a major theme was that a certain topic was mentioned repeatedly or emphasized by the interviewed persons. For example, all physicians spoke about their experiences with unsatisfactory symptom control, and the nursing staff described particularly stressful situations. Minor themes were also coded. These are topics that come up repeatedly but not in every interview. During the coding process, the themes, categories and subcategories were modified and revised. "The analysis was reflectively accompanied by repeated data sessions of the research team (two full professors, one of them a theologist, one senior researcher, three research assistants, three student assistants: 3 male, 6 female)." Fig. 1 is a visual representation of the research process. There was no feedback on the results by the participants in the study.

### Ethical issues

The study was approved by the Ethics Committee of the Faculty of Medicine at LMU Munich (Az-558–15, 30.11.2017). All participants were provided with information both on the project and on data-protection. All participants confirmed that they were willing to participate in the study.

## Results

Three major themes and three minor themes emerged from the interviews and are presented in Fig. 1. Symptom control was at the forefront of the major topics. Particularly in the physicians' statements, symptom control emerged as the dominant topic when unsatisfactory dying processes were described (key for the quotations: PH = physician, N = nurse, first number = number of the interview, second number = paragraph within the interview). In hospices, patients are usually referred to as guests, this is only mentioned in verbatim quotes.

- I. Major themes
  - Theme A: Unsatisfactory symptom control

Physicians speak of an unsatisfactory course of death when symptoms cannot be controlled or can only be controlled deficiently. Often, symptoms were mentioned that can be sorted according to the classic categories of hospice work:

- A-1: Physical suffering: Here, nausea, shortness of breath and pain are mentioned as symptoms that cannot always be satisfactorily alleviated.
- A-2: Psychological suffering: Not infrequently there are patients who.

"cannot develop this calmness at the end of their lives, which the companions can often manage so well, right? The ones who decompensate psychologically at the end, who develop psychiatric symptoms. Who are also negative, aggressive, who are simply, who are people you can't find access to. (N 12, 83)

- A-3: Social suffering: Reticence, fear of rejection or fear of loneliness make the dying process more difficult. Patients are also burdened by the idea that their loved ones could suffer too much. Patients feel abandoned, superfluous, pushed away or to be a cost factor.
- A-4: Spiritual suffering: The fear of being abandoned by God or being punished is mentioned, as well as "life guilt" (PH 9, 49) that burdens the patient.

Physicians speak of an unsatisfactory course of death even when staff and relatives are burdened, for example when someone dies under a "heavy symptom load" (PH 6, 77). A nurse comments: "It is difficult to see someone suffocate when the medication is no longer enough" (N 17, 22). This also applies to imposing symptoms such as bleeding, large wounds, vomiting blood after a fall or restlessness or a tendency to run away in the preterminal phase: A patient in the.

"pre-terminal phase, actually he was bedridden (...) got up, fell, head laceration, a lot of blood, restlessness, and that is, of course, quite unpleasant for everyone involved. And especially when, for example, relatives are present. (...) But, of course, it also puts a strain on the nursing staff, who are of course also directly onsite, right?" (PH 4, 36)

"Personality changes due to brain metastases" (PH 11, 76), for example, are distressing for relatives. This also applies to seizures or severe pain, which is why they often request sedation.

- Theme B: Conflicting goals in symptom control.
  - B-1: Conflicting goals that are difficult to balance.

Physicians speak of an unsatisfactory death when symptom control remains deficient due to conflicting goals. For example, pain is not completely relieved in order to reduce the patient's fatigue or to maintain consciousness. A physician sees.

"the need to preserve the patient's consciousness as much as possible when administering medication but is willing to accept clouding of consciousness to relieve agitation." (PH 8, 28))

Especially palliative sedation does have, as another physician points out, desirable consequences for the professional environment, namely when.

"the patients are simply restless, where you then also have to walk this tightrope between sedation, but still somehow retain their personal rights, that is, their dignity. None of us should be sedated simply because of restlessness. After all, the dignity of care must somehow be preserved." (PH 12, 140)

The conflict between consciousness and self-determination on the one hand and their impairment by pain medication on the other hand can be "solved" by gradually adjusting the appropriate dose. This deliberation takes place even in the extreme situation in which a patient has smeared the environment with his excrement:

"We had a patient who really smeared the room with her excrement, daily. And you have to keep the nursing staff in mind. And to consider this tightrope walk, which restlessness do you allow, which not?" (PH 6, 74)

The challenge also for nurses is to keep freedom from symptoms and pain in balance with the patient's consciousness. It is perceived as difficult when the patient's wishes can no longer be ascertained. One nurse tried to gradually combine autonomy and patient care by giving patients the experience of having better life quality through pain medication:

"And then they try it out and realize they then feel much better, because the quality of life then increases and you are still present, etc." (N 7, 15)

- B-2: Autonomy-emphasizing practice is unsatisfactory:

Patients are often willing to endure pain in order to remain autonomous. Unsatisfactory situations arise when pain medication is refused by patients despite insistent and repeated recommendations from staff, partly out of fear of loss of autonomy or, related to this, fear of loss of consciousness, and in rare cases also the desire to feel pain for spiritual reasons. One nurse notes that it is difficult when patients.

"bear and endure their pain for so long, when there are such good remedies that could make it easier for them, but they just want to endure the pain at that moment, want to or have to, I don't know." (N 16, 27)

From the employees' point of view, patients then make it unnecessarily difficult for themselves and the employees feel helpless. Nevertheless, they want to maintain the patient's consciousness so that they can take the patient's wishes into account. A nurse expresses the following position: If patients refuse symptom treatment because they want to feel their pain, she feels "helpless" (N 19, 37), but she respects the autonomy of the person concerned.

It can be stressful for staff if patients or relatives do not accept the administration of pain medication. A patient does not want to take morphine, but has attacks of breathlessness, leading to unnecessarily difficult situations. (N 7, 81) A nurse remarks:

"So if someone is very restless and wants to get up again and again and falls down again and falls down again, then maybe you try to get him a bit calmer with medication, but sometimes the relatives don't want that. The patient may not want that and then we really have to live with the fact that he falls down four times a day. And that's a bit of a problem, you can't give them anything, no, they don't want to. And then, bang, he's lying on the floor again. A wound here, a wound there. That's sometimes difficult with people who are confused and have a tendency to run away." (N 17, 57)

But there is also an opposing tendency. One physician comments:

"If you reduce opiates, it's actually often the case that at the end of life, metabolic processes tend to lead to a relative overdose, and you then have to gradually reduce a little. This is sometimes difficult to discuss with patients because they are afraid that something will be taken away from them. And they don't see the benefit of less tiredness or less nausea or less constipation." (PH 2, 18)

Sometimes patients also want a higher dosage of painkillers, even though their pain is already sufficiently controlled, simply to cloud their consciousness.

Respect for patients' autonomy must respond to very different trajectories, which poses a particular challenge for nurses: Some patients simply wish to be able to sleep and "cross over", some wish to remain "present" and be able to "witness" (N 11, 15) and "reflect" on their dying. (N 19, 18) The nurse who expressed this would argue more for presence, admittedly not in a way that would require someone to "then endure pain". (N 14, 23).

A nurse tries to educate patients who want to endure their pain about symptom control options, but ultimately accepts the person's wish. (N 22, 41) In addition, there is a tendency to "dampen down" agitation (N 7, 50), although it is not always clear what the cause of the agitation is. Therefore, one would have to look more closely at what is important for each individual at that moment. There is a danger that "routines" (N 19, 11) creep in, which do not do justice to the patient or his individual well-being. Pain and symptom control is named by one nurse as a prerequisite for a calm and thus good death. At the same time, she is aware that it is not simply a matter of "subduing" agitation (N 5, 22), but of looking more closely at where the patient's restlessness specifically comes from and how it should be treated.

- B-3: Practice that overrides patient autonomy:

Behind some patients' desire to endure pain, there appears to be a certain notion of autonomy and consciousness. In individual cases, an autonomy overriding practice seems to be indicated, although the extent of this violation to the patient’s autonomy remains unclear or extremely small. One physician notes that successful symptom and pain control is not always easy, for example when a patient refuses to take painkillers:

"If she had taken her painkillers, she would have been pain-free. But because of her psyche structure, you had to talk to her every day with the tongues of angels. Until she was so somnolent at the end that they could give her a Perfusor, then there was good symptom control, then she also died peacefully, peacefully, so she died quietly." (PH 14, 19)

Here, the patient's wishes were considered for a long time, but were overruled in the end.

Another nurse remarks:

"Or if someone, well, can no longer assess his situation at all, then I also inject him with something if he, that is, if I have the feeling that it is necessary. I mean, of course, I also have an on-demand medication here. I don't just do something out of nothing or anything like that, right? But there is a situation where you can't always ask someone who has shortness of breath and is already on the way: May I inject you with morphine now? I always tell them what I'm doing. Right? I'll go now and get morphine, or: I'll be right back with you. I see you're having shortness of breath; I'll inject you now, okay? That's how I would do it." (N 3, 44)

Here it remains unclear whether this respects the patient's autonomy or not.

In another case, a patient wanted less medication, which was not granted, leading to her death from an overdose. In the case of prefinal agitation, medication is given as needed to help patients achieve a state of calm. The statement by a nurse leaves the question open of whether the administration of medication to calm patients overrides their autonomy or not:

Apparently restlessness contradicts the idea of a good dying process, as a nurse comments: "That is also a symptom, this restlessness, and often we know with this restlessness that it is a prefinal restlessness, and then we give what they need, and then they become peaceful right away. And we’re generous with this. So, if we now give them something and they aren’t calm after ten minutes, then we directly give something again. So, we are allowed to very quickly give them what they need again." (N 26, 25)

Due to the risk of falling, one patient was sedated because there was not enough staff available at night to monitor the patient. A nurse remarks:

"I don't know, the ALS patient we had the other day, going back to the issue of dilemmas, which ’I still see now, who, so to speak, gets multiple midazolam tranquilizers because for us’, or because we don’t allow her to, or we know that she is restless because she has to go to the toilet and she is incontinent and she feels the need to urinate and has to go to the toilet, but it’s just not possible - to somehow take this urge to urinate away because we can’t take her out alone during the night shift. That’s why she is sedated. That’s difficult for both sides, of course, because as a caretaker, you don’t want to ruin your back doing that, but at the same time you can’t be responsible for not fulfilling a patient’s need by sedating them, which is very tricky.” (N 7, 36)

Even in situations in which the possibility of self-endangerment is seen, a practice that overrides patient autonomy likely to occur.

"These are situations that we don’t cope well with. We have to manage it somehow, and then it’s usually just a case of giving midazolam, not us, the physicians. And that there’s no other way because it’s no longer controllable. Or someone who wants to endanger themselves. These are of course limits where you say: We won’t allow that here." (N 18, 30)

In the opinion of a nurse, the common practice is not questioned enough,

"there is always the medication that is used – it hardly differs. And we give that then. Because that's what has been ordered. In case of nausea, we give them the same thing, and whether someone then reacts to it in such a way that he sleeps for twelve hours, and the other person, however, only sleeps for two hours, the main thing is that the nausea is eliminated." (N 14, 44)

- Theme C: Conflicts.

Conflicts arise when relatives demand therapies that physicians refuse because they are not indicated. For example, as one physician reported,

"The wife here called the emergency physician when her husband had the slightest problem, which is completely contrary to the hospice idea, and she demanded treatments. ... I then explained why the treatment was useless. I think it was about infusion therapy. And I explained why it wasn’t good. And she said: 'Yes, I understand. But I know it’s helping him.’ We didn’t come to a consensus." (PH 3, 43)

The same physician notes that he was once "very harshly accused" of "letting his father die of thirst and torturing him and sending him to his death in a very unfortunate way.” (PH 3, 43) The physician.

"was ultimately only able to resolve the situation by hanging up an infusion and letting it drip so slowly that it ultimately had no effect at all. But of course I failed in my arguments. I didn’t get through to them. So that wasn’t good." (PH 3, 57)

Another physician reported that relatives even wanted to force therapy:

"And relatives would always stand in the doorway in front of the nurse and threatened to call the police and use physical violence. That was a troubled dying process." (PH 4, 58)

In another case, relatives demand sedation for a patient with only mild symptoms. The physician refuse and is met with hostility by the relatives. In some cases, it is reported that relatives give the patient food, even though this s harmful to him.

Occasionally, relatives do not want therapy, even though it is considered indicated by the physicians:

"I think a medication was given that they didn't want to be given. It could have been something like that, yes. And then they called the police, yes. Or because they thought we were torturing the residents." (N 2, 65)

We also observed conflicts between patients and relatives, especially concerning potential therapies. One woman.

"every ten minutes actually accused the nurses of killing her husband. And the man wanted painkillers. So that was clearly not what the patient wanted. And then, the wife then called the emergency number several times. So, it was a difficult situation. It couldn't be resolved." (N 18, 37)

Sometimes relatives use violence if they have been treated badly all their lives. Conflicts within a family can increase pain and anxiety in patients, as physicians have repeatedly stated.

- II. Minor themes.
  - Theme A: Repressing an illness.

Not dying well means completely repressing an illness, denying it:

"And patients who you can tell are not ready to die. That somehow, as I said before, they haven't finished yet. Still holding on to something. I just find that, that's difficult." (N 27, 61)

Some patients completely suppress their suffering and dying:

"Or now recently we had a patient who simply didn't let anything be done to her. Nothing at all. With a large wound. And who was always convinced that if you don't do anything, you negate its existence, so to speak. If you don't do anything, then there is nothing. And who also died with this denial." (PH 12, 43)

Other patients want therapy because they still have hope and are disappointed when nothing more is done. Staff feel helpless in such situations.

According to a nursing director, whether a death is peaceful or not is related to the issue of processing or repressing an illness: If the illness is pushed away until the end, this is "difficult to bear" for all involved, thus especially for the nursing staff. (N 24, 31)

When the conscious confrontation with the illness or the acceptance of the illness does not take place, it is manifested as restlessness:

"There were already many cases who really did not want to talk about their illness, neither with the family, nor with anyone, and who were then simply extremely restless and no longer responsive, where one then tried to really give something sedating and painkillers and they needed much higher doses than with other patients to accompany the symptoms." (N 28, 49)

However, there is also the opposite attitude among staff, who show understanding for patients to not want to accept their death:

"And I therefore also accept when there are guests here, because there are also guests who do not want to accept it until the end. And that makes me angry when they have to. (...) But I would say that most of them cannot. And that something is interpreted into it. That somehow they should. They're supposed to be able to. Because they are here." (N 9, 34)

A patient is shielded by relatives who give her hope, and the staff lose access to her.

- Theme B: Course of time.

The course of death is perceived as unsatisfactory if it takes too long and is agonizing or comes surprisingly quickly. Whether the course of death was good or bad depends on the point of view and is judged by the feeling of whether it was right or appropriate, as one nurse explains:

"For me, it is quite often the case that I somehow decide based on my feelings. I can't say, for example, that we had a woman who quite suddenly, quite unexpectedly for all of us, just died. And now you can say, oh, that was good for her, because she had such terrible wounds, and she didn't have to, well, that was somehow good. But for me it doesn't feel so good. So, I don't know. And then there are other guests who are really prefinal and you have the feeling that everything drags on insanely long and yet I have the feeling that in the end it somehow fit, yes. (...) It must also be right from the feeling, and it must also, you must also feel that it was somehow right for the guest." (N 2, 90)

Staff feel helpless in such situations, they seem to have failed:

"I've experienced a situation like that. It happened so quickly, the on-demand medication couldn't take effect that quickly, even though I administered it immediately. In that situation, the patient choked on his blood, and he was fully conscious, he was completely aware of what was happening." (N 29, 18)

This can also be difficult for relatives. It is also perceived as unsatisfactory when a patient is found dead because nothing could be done.

"One thing is when we find someone dead. It's always a bit like, we weren't there. And it's a recurring theme, even in the handover. Yes, then we found him dead, I say, okay, then he died alone. --- is perceived as a failure." (PH 12, 35)

- Theme C: Truth at the bedside.

An unsatisfactory course of death is described when a patient felt deceived about her state of health, i.e. lied to, and died "disappointed, bitterly disappointed". (PH 3, 21) A case is also mentioned in which a patient deceived the physicians by double-crossing them with opioids, thereby destroying the trust in their relationship.

Sometimes patients deny their illness until the end so that physicians are unable to communicate with them.

"But of course there are many, sometimes even the young patients, who deny that they are ill, even completely deny they are dying. I've seen that very often, and it's sometimes very, very difficult for us to deal with, because then it's often the relatives who have the problem, who have never talked about it and often suppress the illness. And it's only at the end that they are blindsided by how badly their relative is perhaps doing." (PH 7, 37)

## Discussion

In literature, an unsatisfactory death is often associated with (1) inadequate symptom control, (2) poor communication with patients and relatives, and (3) an unsatisfactory dying process for staff (too fast, too slow, patients in denial), whereby the event of death is emphasized over the dying process ([12], similarly [11], who provide an overview). This is plausible for dying processes in hospitals. In our study, which took place in hospices and palliative care units, the focus was, again understandably, on the *process* of dying. In particular, (1) the *autonomy* of patients, (2) the *needs of staff* and (3) *conflicts with relatives* came to the fore in addressing symptom control.

The interviews show typical ideas of dying well but articulate descriptions of dying badly as well, which are more specific, often refer to concrete cases that are remembered, and provide information about the gap between the ideal and reality.

Observations of unsatisfactory courses of death are related to deficient symptom control (A) [12]. Other observations relate to conflicting goals in symptom control: In some cases conflicting goals are difficult to balance (B-1), in some cases the patients themselves set limits to medical and nursing measures in order to preserve their autonomy (B-2), and in some cases the staff set limits in order to protect themselves from the stress caused by difficult courses of death (B-3), [17] even though this may compromise the autonomy of the patients.

If the dying process becomes a burden for the surroundings, an autonomy-overruling practice may become established which, we assume, is not or not always perceived or reflected upon as such: Patients are sedated or urged to take medication. In the interviews it was expressed that clouding of consciousness was accepted in the case of pain medication without having asked for the patient's wishes, in cases where especially the restlessness of the dying person and his or her behavior became difficult to bear for the surrounding people. In some cases, there are grey areas between patient care and autonomy or situations that do not show a clear allocation but nourish the suspicion of a practice that overrides patient autonomy. Here, staff obviously have different ideas about a satisfactory course of death than the patients themselves. However, staff also point out that the causes of the perceived difficult patient behavior should be questioned before resorting to routine and possibly autonomy-overriding sedative measures. What is therefore criticized in the interviews is that sedative medication can establish a routine that does not contribute to the individual patient's well-being. Nevertheless, the tension between enabling a successful symptom and pain control on the one hand and enabling a conscious and therefore autonomous death on the other hand, is regularly pointed out: It is true that clouding of consciousness should be avoided as much as possible, so that physicians and nurses can communicate with the patient and in this way discover his wishes.

The interviews even refer to the abilities of the employees and, above all, the limitations of such, and additionally reveal the needs of the employees, which are generally less emphasized than the needs of the patients. We conclude that the needs of staff have an influence on patient treatment that should not be underestimated: If staff are overly stressed by restlessness, they tend to sedate patients, even if such a medical intervention is not in accordance with the patient's wishes. If, on the other hand, the aim is to explore the patient's wishes and get to know their needs, there is a tendency to discontinue mind-altering medication, even if this is not in accordance with the patient's wishes.

It is obvious that family members are burdened during difficult death processes. It is worth noting, however, that such burdens can have a reciprocal effect on the staff and lead to conflicts: The relatives sometimes have different ideas of a satisfactory death than the patient or the staff. In some cases, conflicts also arise with relatives that cannot be resolved (C).

Minor topics are mentioned regularly, but not continuously: an unsatisfactory death is mentioned when patients repress their illness, when the dying process seems too fast or too slow (in both cases the helplessness of the staff is discussed), or when the trust relationship between staff and patients is harmed or destroyed.

## Conclusion

Ideas of a satisfactory death are often idealizations. An idealization effects the perception of a death process that does not correspond to an ideal. In other words, normative expectations of a satisfactory death evoke the observation of unsatisfactory courses of death. If these expectations are set (too) high, the perception of an unsatisfactory death becomes more likely. The results of our study confirm the opinion expressed in literature that nursing staff in particular regard medically and socially monitored deaths as being satisfactory, [8, 14] while deaths that do not meet these expectations are qualified as unsatisfactory.

Physicians, nurses and volunteers talk about unsatisfactory courses of death because, we assume, the practice in hospices and palliative care units is based less on standardized medical and nursing procedures than in hospitals and the number of clear routines is comparatively low. As a result, there is a need for those involved to get to know a patient as well as possible (which is often not possible due to the short length of stay), to listen to their needs and wishes again and again, also in case their ability to communicate diminishes or disappears completely. This should enable medical and, above all, nursing measures to be better adapted to the different needs and wishes of dying people.

If many deaths are described as being satisfactory by the social environment, we assume the few unsatisfactory deaths (at least the ones perceived as such) create a need for explanation and justification. Because standards and routines only partly determine the actions of employees, those involved need to have a feel for the requirements of a particular situation. We therefore differentiate between *situational demands* and *ethical principles* that play an implicit role in the interviews, above all the principles of autonomy, no-harm and beneficence.

It is undisputed that this describes ideals that cannot or can hardly ever be fully realized in reality.

Our empirical findings show that situational demands that cannot be considered simultaneously can compete or even conflict with each other. (a) If the aim is to achieve a reasonable *balance* between competing demands, having a sense for the right action in a situation should provide a sufficient foundation. The situation of a (b) *conflict* between different situational demands is more problematic. Here, the binding power of situational demands is, at least in our view, inconceivable without consideration of ethical principles.

We distinguished between two types of conflict: Firstly, situational demands can come into *conflict with each other*. In this case, it must be justified why one demand should be given clear preference over another. Secondly, within such a practice of favoring situational demands, there may also be a *conflict with an ethical principle*.

In the literature the objection is raised that *principlism* employs, with its four principles of respect for autonomy, justice, nonmaleficence, and beneficence, universal ethical principles which are schematically applied to concrete individual cases [18]. Beauchamp and Childress present an alternative conception. To recognize the moral dimension of a situation and ascertain what should be done, and to justify actions (that is, decisions) before oneself and others, are two different activities (though not, perhaps, fully independent from one another). They can be distinguished by the mere fact that justification, according to principlism, only becomes necessary in the case of conflicting principles [19]. In all other cases moral experience in general is a "credible and trustworthy" source of ethical knowledge [20].

We advocate therefore a principle-based approach which, of course, leaves room for situational intuition, namely where principles do not do justice to the specificity of concrete cases. The need for justification thus lies in forming an intuitively plausible hierarchy of competing or conflicting situational demands in the respective context without violating an ethical principle. It is worth noting, and yet little illuminated in the literature, how principles such as beneficence or nonmaleficence on the one hand and the principle of autonomy on the other relate to each other in the event of conflict if there is no established hierarchy, even when such a conflict is about the needs of the employees, i.e. not just the needs of the patients. If several ethical principles are violated, i.e. if ethical principles conflict with one another, a pluralistic model, at any rate, would not provide a rule for resolving such a conflict. However, as long as the patient is capable of autonomy, the principle of autonomy may not be subordinated to other principles. In this respect, even if one advocates a pluralism of principles, it seems reasonable to assign the principle of autonomy a special position above other ethical principles. It is not clear from the interviews whether a patient's autonomy was actually overruled. We therefore do not trivially formulate hypothetically and ethically: If a patient's autonomy were overruled, the otherwise noteworthy demands, in particular the demand to enable a peaceful death, could not provide any legitimate reasons for such an autonomy-overruling practice.

Finally: In our eyes, interpretive descriptions (a patient dies "too quickly" or "too slowly") form narratives, and appear less problematic unless they are intended to provide reasons for action.

### Practical implications

In addition to the needs of patients, the needs of employees deserve special attention. Conflicts between different situational demands and conflicts between situational demands and ethical principles also require careful attention.

### Limitations

Asking only professionals inevitably leads to staff perspectives. Future research could include the perspective of patients and relatives.

### Supplementary Information

**Supplementary Material 1.**

## Data Availability

The datasets generated and/or analyzed during the current study are not publicly available due to ensuring data protection and anonymity for the interviewees but are available from the corresponding author on reasonable request.
